# Insect Consumption Attitudes among Vegans, Non-Vegan Vegetarians, and Omnivores

**DOI:** 10.3390/nu11020292

**Published:** 2019-01-29

**Authors:** Anna-Liisa Elorinne, Mari Niva, Outi Vartiainen, Pertti Väisänen

**Affiliations:** 1 School of Applied Educational Science and Teacher Education, University of Eastern Finland, FI-80101 Joensuu, Finland; outivar@gmail.com (O.V.); pertti.vaisanen@uef.fi (P.V.); 2 Faculty of Educational Sciences, University of Helsinki, 00014 Helsinki, Finland; mari.niva@helsinki.fi

**Keywords:** insect consumption, vegetarian, vegan, omnivore, attitude, intention, theory of planned behavior

## Abstract

Abstract: Background: Consumption of foods of insect origin is encouraged, since insect consumption is seen as one of the responses to the environmental impact of meat production. This study examines the attitude (A), subjective norm (SN), perceived behavioral control (PC), and food neophobia (FN) toward the consumption of foods of insect origin, as well as the conditions for eating insect-based foods among vegans, vegetarians, and omnivores. Methods: The data was obtained by using an online survey and convenience sampling (*n* = 567, of whom omnivores represented 74%, vegans 5%, and non-vegan vegetarians 22%). Results: The three dietary groups exhibited significantly different intention (I) to eat foods of insect origin. Vegans held the most rigid negative attitude (A), and their subjective norm (SN) to eat insects was weaker compared to that of omnivores (*p* < 0.001) and non-vegan vegetarians (*p* < 0.001). Vegans’ perceived behavioral control (PC) over their eating of insects was stronger compared to that of omnivores (*p* < 0.001) and non-vegan vegetarians *p* < 0.001), and they were more neophobic than omnivores (*p* < 0.001) and non-vegan vegetarians (*p* < 0.001). Non-vegan vegetarians held the most positive attitude toward eating insects, and both non-vegan vegetarians and omnivores thought that insect consumption is wise and offers a solution to the world’s nutrition problems. In contrast, vegans regarded insect consumption as immoral and irresponsible. Conclusions: Vegans’ weak intention, negative attitude, and low willingness to eat insects in the future exhibit their different dietarian identity compared to that of omnivores and non-vegan vegetarians.

## 1. Introduction

Foods made from insects have a low ecological footprint, and due to their high nutrition content, they serve as a potential sustainable supplement to the existing protein sources for human nutrition [1]. Consumers’ willingness to eat foods of insect origin is, however, weak [2], and varies between different insects [1]. The negative attitude is explained by Western food culture traditions, in which insects have not been classified as a source of food. Instead, the majority of consumers regard them as rather disgusting and inappropriate for human nutrition [1].

Vegetarians form an interesting and less studied population group with regard to insect consumption. Supposedly, vegetarians’ attitude toward insect consumption varies depending on what role animal-derived foods have in their diet and how they see insects as sentient beings in the world of edible animals [3,4]. In this respect, vegans are more restrictive than non-vegan vegetarians [5]. Vegetarians can be seen to form a continuum of different groups with a progressive degree to which animal foods are avoided. Beardsworth and Keil [6] classified their respondents into six types: type I vegetarians occasionally ate red meat and poultry, type II vegetarians avoided consuming red meat and poultry, but ate fish, type III vegetarians also avoided fish, but ate eggs, type IV vegetarians also excluded eggs, but ate dairy, type V vegetarians also excluded dairy products, except for rennet-free cheese, and type VI vegetarians (i.e., vegans) avoided all animal-derived products [6,7]. In this article, the term “vegetarian” is used as a general concept referring to all kinds of vegetarian diets. When a distinction is made between vegans and other vegetarians, the latter are referred to as “non-vegan vegetarians”.

However, the boundaries between different categories are not stable, and people may shift from one diet to another [6,7]. In addition, non-vegan vegetarians and vegans differ from each other, not only in their food selection, but also in their motives to adhere to a vegetarian diet [7,8,9,10] and in their dietarian identity profiles [11]. Non-vegan vegetarians’ and vegans’ attitude toward meat also differs from that of omnivores [7]. All these factors are likely to exert their effects on both non-vegan vegetarians’ and vegans’ intentions to accept foods of insect origin into their daily diet.

In their study on consumer acceptance of mealworm products in the Netherlands, Tan et al. [12] speculated that ”vegetarians may actually be open to considering insects as food”, although vegetarians were not in particular focus in their study. In a qualitative study among Dutch consumers of insect-based convenience foods, House [4] found that vegetarians may eat insect-based foods for environmental reasons, but also because insects are seen to lack sentience or capacity to suffer, or because they are not regarded as “proper” animals. House’s informants included different types of vegetarians, along with people who consumed no other animal-based foods apart from insects. To our knowledge, House’s study is the only earlier research focusing on vegetarians’ attitude toward insect consumption and their use of insects-based foods. 

### 1.1. Motives to Adopt a Vegetarian Diet

Several studies have identified a variety of motivations for adopting a vegetarian diet. The main three categories of reasons are health issues, ethical issues, and environmental concerns [7,8,9,10,13,14]. The initial motivation to adopt a vegetarian diet may vary among non-vegan vegetarians and vegans; however, it is typical that when the “vegetarian career” proceeds, the scope of reasons is broadened by linking new concerns to earlier ones [4,7,8]. Vegetarians’ different motives form a trajectory, which can be seen in their dietary pattern. For instance, their dietary choices may develop from eating organic food to partial vegetarianism, or through ovo-lacto-vegetarianism to veganism [8]. 

Initial motives to adopt a vegetarian diet varies between non-vegan vegetarians and vegans. Characteristically, vegans are more concerned about animal welfare and the environment. They have feelings of aversion towards meat and they justify their dietary choice more commonly by expressing ethical reasons [7,11]. This was also found among Finnish adult vegans [10], whose reasons for transitioning to a vegan diet stemmed mostly from a concern of animal rights and environmentalism. Reasoning their dietary choice, vegans argued that killing and intensive farming of animals is wrong and that animal farming increases the environmental burden. They also felt disgusted by the taste of meat [10]. 

### 1.2. Attitudes toward Meat

Previous research suggests that omnivores and vegetarians have different stances toward meat consumption [7]. Vegetarians link meat consumption with cruelty, killing, disgust, and poor health, whereas omnivores associate meat consumption primarily with luxury, status, taste, and good health [7], and rationalize it as natural, normal, necessary, and nice [15].

Demographics, such as gender, education level, income, social class, age, and ethnicity have been shown to influence meat consumption [7]. Income and age have been shown to be positively correlated to a preference for meatless meals and inversely related to a preference for red meat [16]. In Latvala et al.’s [17] study, it was shown that the consumer cluster with the most established meat consumption was male-dominated, while the cluster which was in the process of reducing meat consumption was dominated by women, the highly educated, and those with higher incomes. Vainio et al. [18] suggest that natural concerns and health and weight control motives are important in maintaining and adopting a plant-based diet, whereas convenience and price motives were barriers to substituting plant proteins for meat. 

Taking into account the considerable differences in the use of different kinds of animal-derived foods in different vegetarian diets, it remains to be shown how and to what extent non-vegan vegetarians and vegans are ready (or willing) to accept insects into their diet and how they might differ from each other and omnivores in this regard.

### 1.3. Dietarian Identity

Earlier studies show that beyond food selection, vegans and non-vegan vegetarians also differ from one another in their dietarian identity (i.e., how they think, feel, and behave with respect to their diets) [7,11]. Characteristically, vegans see their diet being more interwoven with their identity. They also feel more positive toward other vegans, and are more supportive of a vegan lifestyle compared to non-vegan vegetarians’ commitment to a non-vegan vegetarian lifestyle. However, they are more prone to judge adherents of other dietary groups and experience other people judging them more negatively for their diet. Generally, vegans have stronger motives to consume their diet strictly compared to non-vegan vegetarians [11]. 

Being vegan is more than just adhering to a meat-free diet. It is an ideology, a way of life, and a value perspective for all decision-making [7,8,10,11]. In coping with their daily food management, vegans have developed different strategies [10]. Characteristically, vegans study package labels carefully and boycott food if necessary, for example, genetically modified foods (GMO foods). Many vegans also carry their own foods with them in different situations [10]. Partly due to these dietary strategies, vegans and sometimes also non-vegan vegetarians face more challenges in social life [10,19,20]. For example, in the study of Elorinne et al. [10], adult Finnish vegans experienced other people contemplating the nutrition value of their diet, and social gatherings often caused embarrassment or uncomfortable situations for them. 

Vegans may further differ from each other in their compliance and agency related to veganism. Based on their study, Larsson et al. [14] classified Swedish adolescent vegans into three groups: conformed vegans, who were not so committed in their veganism, organized vegans, who were strongly anchored in vegan ideology, and individualistic vegans, who were committed to their dietary choices, but felt no need to join and associate themselves with other vegans. 

### 1.4. Consumers’ Willingness and Intentions to Eat Foods of Insect Origin

Prior research has suggested that consumers’ acceptance of insects as food is affected by the cultural and social context, whereas various psychological, situational, and sociodemographic factors (sex, age) have been found to be associated with consumers’ willingness to adopt insects as a food source [2]. The likelihood of accepting insects as food seems to increase with consumers’ awareness of the environmental impact of food production [21]. In a recent research study, Menozzi et al. [22] also showed that consumers’ curriculum of studies was a significant factor affecting the acceptance of food of insect origin. Students enrolled in programs of food and environmental science-related curricula exhibited higher intention to consume foods of insect origin than students of social sciences. Generally, western consumers don’t regard foods of insect origin as a delicious and nutritious option, instead insects are seen as a necessary response to overpopulation and the environmental concern caused by meat production [22]. However, in a recent study [23], consumers of insect and vegetarian products were defined very positively. People regarded them as more health-conscious, environmentally friendly, imaginative, brave, interesting, and knowledgeable than meat eaters.

In several studies, the rate of neophobia and the sense of disgust have been suggested to be relevant predictors of acceptance of insect consumption [2,24,25]. Research also shows that a low level of fear of new foods (food neophobia) increases the willingness to eat foods of insect origin [26,27]. According to Modliska and Pisula [28], personality traits have an impact on food neophobia, and people who are prone to feel disgust are likely to be more neophobic than others. Generally, products of non-animal origin trigger lower levels of food neophobia than animal-based products [29]. With regard to insect consumption, recent research has shown that consumers make a distinction between different kinds of insects; some are better accepted than others [2]. 

In this research, we examine Finnish consumers’ intentions to consume foods of insect origin in the near future in three different dietary groups (omnivores, non-vegan vegetarians, and vegans). As a theoretical frame, we use Ajzen’s [30] Theory of Planned Behavior (TPB). According to the theory, individuals’ intentions to change their behavior determines the possible change in actual behavior. Intention consists of three main factors: consumers’ attitude (A), subjective norm (SN), and perceived behavioral control (PC). If the intended behavior change is expected to be beneficial, if there is social pressure to change the behavior, and if there are no obstacles to adopt the intended behavior, then individuals are more capable of changing their behavior.

To our knowledge, in the context of consumer acceptance of insect consumption, so far only Menozzi et al. [22] have utilized this theory in their analysis of the consumption of novel food (chocolate) made from insects among young Italian adults. The TPB model accounted for 78% of the variance in intention and 19% of the variance in behavior. Consumers’ attitude and perceived behavioral control were statistically significant predictors of intention, while intentions and perceived behavioral control predicted behavior. By contrast, respondents’ subjective norm was not a significant factor in forming the behavioral intention. Menozzi et al. [22] concluded that social norms would be more predictive in the case of adolescents than among adults.

Vegetarian diets are popular, especially among Finnish adolescents, although young people’s vegetarianism has been proved to be short-term and flexible [31]. The large share of non-vegan vegetarians and vegans in our sample provided us with an opportunity to examine both non-vegan vegetarians’, vegans’, and omnivores’ attitudes toward insect consumption and their intentions to consume or not to consume foods of insect origin. 

The aim of this study is to examine how different dietary groups (vegans, non-vegan vegetarians, and omnivores) differ from each other in their attitude (A), subjective norm (SN), perceived behavioral control (PC), and food neophobia (FN) toward the consumption of foods of insect origin. In addition, we examine under what conditions insects could be used and what the main reasons are for eating and not eating foods made of insects. Based on previous research and the theoretical frame, we set the following hypotheses: H1: Vegans, non-vegan vegetarians, and omnivores differ from each other in their A, SN, PC, and FN towards the consumption of foods of insect origin; and H2: Vegans show weaker willingness to consume foods of insect origin than non-vegan vegetarians and omnivores do.

## 2. Subjects and Methods

### 2.1. Data Collection

Participants were recruited as part of a larger online survey concerning Finnish consumers’ intentions to eat foods of insect origin in the near future (Vartiainen et al. submitted). The data was obtained by convenience sampling and using a structured self-administered questionnaire. The request to respond to a survey on insect consumption was delivered by using social media (https://twitter.com/yletiede) and researchers’ Facebook pages, as well as the digital versions of one national (https://www.maaseuduntulevaisuus.fi/) and one metropolitan area (https://www.city.fi/) newspaper. Altogether, 567 consumers responded. Out of these, 27% were some kind of vegetarian (*n* = 150, of whom 64% semi-vegetarians, 17% lacto-vegetarians, 2% lacto-ovo-vegetarians, and 17% vegans). In total, 22% were non-vegan vegetarians and 5% were vegans.

### 2.2. Measurement

The first section of the survey comprised demographics and background information (age, sex, education level, place of residence, cooking habits, special diets, and earlier experience of eating insects). The second section comprised the Food Neophobia Scale [32] (Pliner and Hobden 1992), and the third and fourth sections focused on planned behavior constructs (attitude, norms, behavioral control) and intentions. In the end, participants were asked to explain the reasons for their intentions to eat or not to eat foods of insect origin by using an open-ended question.

Respondents’ fear towards new foods (FN) was originally measured by 10 statements. The attitudes (A) towards foods of insect origin were asked about by using 30 statements, subjective norms (SN) in food behavior by using 14 statements, perceived behavioral control (PC) over their intended behavior either to eat or not to eat foods of insect origin with 11 statements, and intentions (I) to consume foods of insect origin by using 17 statements. All statements were presented using a Likert scale (1 = totally disagree, 7 = totally agree). 

### 2.3. Data Analysis 

The data was analyzed by using SPSS Statistics Version 23. Some variables were reversed so that they measured respondents’ answers to a similar direction. To uncover and identify the underlying relationships between the measured variables, eight constructs were built based on the internal consistency of the variables and partly on researchers’ a priori hypotheses. Cronbach’s alphas of the constructs varied from 0.756 to 0.970 (Table 1). A new variable, “food neophobia” (FN), summing up the scores of six of the original variables was constructed, and this summated score measured the level of food neophobia for each respondent. Higher scores indicated higher neophobia. Since vegans intentionally avoid foods of animal origin, some items in the original FN scale were excluded due to the fact that their inclusion would have painted an incorrect picture of vegans’ neophobia. The excluded variables were: “I am fond of foods from different countries”, “Foods from different countries look too odd to eat”, “I am very selective in my eating” and “It is fun to try foods, which represent different food cultures”. The six remaining items included in this study are shown in Table 1.

The respondents’ perceptions of conditions for consuming foods of insect origin were operationalized as Healthiness, Safety and Convenience, and price. Healthiness and Safety both consisted of three statements about respondents’ perceptions of health (H) and safety (S)-related conditions for consuming foods of insect origin. Convenience and price consisted of four statements concerning the convenience and cost (C)-related conditions for consuming foods of insect origin. (Table 1). These constructs were used in statistical testing (Chi-Square test, One-Way ANOVA). A Bonferroni post-hoc test for multiple comparisons was used in analyzing the differences between different dietary groups. *p* ≤ 0.05 was considered statistically significant. Eta square was used as an effect size index in a one-way ANOVA and Cramer’s V in a Chi-Square test. K-means cluster analysis was used to group respondents based on their planned behavior (A, SN, PC, and I), and perceptions of conditions for the consumption of foods of insect origin in terms of the three summated scale variables Healthiness (H), Safety (S) and Convenience and price (C). Univariate ANOVA confirmed that these variables functioned well in classifying the respondents into different clusters (*p* < 0.001).

## 3. Results

### 3.1. Demographics

In total, 567 consumers filled in the questionnaire. Out of these, 67% were women (*n* = 379). There was good representation across the different age groups (Table 2). Out of the respondents, 73% were omnivores (*n* = 417), 22% were non-vegan vegetarians (*n* = 124), and 5% were vegans (*n* = 26). The group labeled as non-vegan vegetarians merged together semi-vegetarians (*n* = 97), lacto-vegetarians (*n* = 25), and lacto-ovo-vegetarians (*n* = 3). 

Based on their sociodemographic characteristics (sex, age range, education level, and place of residence), the respondents were differently distributed in terms of the three dietary groups of omnivores, non-vegan vegetarians, and vegans (Table 2). A large majority of men were omnivores, while among women, the share of omnivores was 16 percentage points lower, and the proportion of non-vegan vegetarians and vegans was higher among women than among men. Of the eldest group, almost nine out of ten were omnivores and relatively few were non-vegan vegetarians or vegans. In the younger age groups, 64–75% were omnivores and 20–31% were non-vegan vegetarians. The share of vegans was 4–6% in all age groups. Among those with the highest education (licentiate or doctoral education), the share of non-vegan vegetarians was as high as 38%, and among those with another academic degree as high as 25%, whereas among those with basic or secondary education the share was 15–16%. However, among those with comprehensive school education, the share of vegans was higher than in other educational groups. Among city dwellers, there were more non-vegan vegetarians and vegans than among those living in rural areas. 

### 3.2. Attitude and Planned Behavior toward Insect Consumption

The results show that omnivores, non-vegan vegetarians, and vegans differed significantly in their A, SN, PC, and FN (Table 3).

Characteristically, vegans deviated significantly from the adherents of other dietary groups in their attitude towards insect consumption. Pairwise comparisons with Bonferroni adjusted *p*-values showed that there were significant differences in attitude (A) toward insect consumption between vegans and omnivores (*p* < 0.001), between vegans and non-vegan vegetarians (*p* < 0.001), and between omnivores and non-vegan vegetarians (*p* < 0.05).

Table 4 shows in more detail how, and in which respects, the three dietary groups differed from each other in the various aspects of planned behavior and food neophobia. Non-vegan vegetarians’ attitudes were the most positive, and they agreed the most with the following statements (but for many statements they did not statistically differ from omnivores): “insect consumption can be a solution to the world’s food problem,” “using insects in food production is a good thing,” “the entering of foods of insect origin into the market would be a good trend,” “the use of insects should be promoted in food production,” “the eating of insects is wise,” “the use of insects as human food should definitely be approved in Finland,” and “I want to be a responsible consumer and to eat insects since I know that eating them is sustainable” (Table 4). 

Vegans agreed the most with the following statements: “insects should not be used in food production,” “foods made of insects are a bad thing,” “I want to be an ethically responsible consumer and I don’t want to utilize insects in my own diet,” and “the use of insects in food production is morally wrong.” In the latter statement, vegans significantly differed from both non-vegan vegetarians (*p* < 0.001) and omnivores (*p* < 0.001).

Vegans exhibited having less social pressure (SN) in their food choice with regard to insect consumption. Pairwise comparisons with adjusted *p*-values showed that there were significant differences in (SN) between vegans and omnivores (*p* < 0.001), and between vegans and non-vegan vegetarians (*p* < 0.001). 

Vegans agreed significantly less than non-vegan vegetarians (*p* < 0.05) and omnivores (*p* < 0.05) with the statement “I could eat foods of insect origin since people close to me think that I follow novel trends in my food selection.” At the same time, they valued the opinion of people that are important to them more highly, since they agreed more than others (*p* < 0.001) that “people that are important to me would respect me less if I ate foods made of insects.” Non-vegan vegetarians agreed the most with the following statements measuring the subjective norm: “people I respect could eat foods of insect origin” and “I could eat foods of insect origin if my friends recommended eating them.”

Vegans’ perceived behavioral control (PC) over their insect consumption was significantly higher compared to that of the adherents of other dietary groups. Pairwise comparisons with adjusted *p*-values showed that there were significant differences in (PC) between vegans and omnivores (*p* < 0.001) and between vegans and non-vegan vegetarians (*p* < 0.001). Vegans agreed most with the following statements measuring behavioral control: “I can easily control that my diet doesn’t contain insects,” “I always watch carefully what I eat,” and “it is totally up to me whether I buy foods made of insects.”

Vegans appeared significantly more neophobic than other non-vegan vegetarians and omnivores (Table 3). Vegans agreed the most with the following statements measuring food neophobia: “if I don’t know what the food contains, I won’t try it” and “I am very selective in what I eat.” In contrast, omnivores agreed the most with “I will eat almost anything.”

In conclusion, in terms of planned behavior, vegans differed significantly from the adherents of other dietary groups. Consequently, their intention to eat foods of insect origin deviated significantly from that of omnivores (*p* < 0.001) and non-vegan vegetarians (*p* < 0.001). 

In addition to the respondents’ intentions, we were interested in how adherents in different dietary groups argued for, or rationalized, their acceptance or rejection of foods of insect origin. When asking about the reasons for consuming or not consuming foods of insect origin by using an open-ended question, we found that vegans were stricter in their attitude toward using insects as food, and they also explained their reasons for being vegans in a detailed manner: 

“Insects are living beings, and it is not right to use them as food. We would need a large number of insect individuals to fill our stomachs, and this is questionable from the perspective of the absolute value of animals. It would also be absurd to adopt insects into my diet after being vegan for many years, since I’ve even avoided animal-derived E-codes (120, 903, and 904). Insect farms are probably not good for the environment either; a swarm of crickets might escape and eat the crop.” 

“I don’t eat any animals as long as it’s possible to select something else to eat. Killing someone (whether it’s larvae, chicken, or dog) just for fun does not fit into my worldview. In addition, the consumption of insects in western countries does not solve the shortage of food in the world when edible food is thrown away.” 

### 3.3. Willingness to Consume Foods of Insect Origin

Vegans were significantly more determined than others that they would not eat foods of insect origin, even if they were nutritious, safe, affordable, and convenient (Table 5).

Pairwise comparisons with Bonferroni adjusted *p*-values showed that there were significant differences in (H) between vegans and omnivores (*p* < 0.001), and between vegans and non-vegan vegetarians (*p* < 0.001), in (S) between vegans and omnivores (*p* < 0.001), between vegans and non-vegan vegetarians (*p* < 0.001), and between omnivores and non-vegan vegetarians (*p* < 0.05, *p* = 0.014), as well as in (C) between vegans and omnivores (*p* < 0.001), and between vegans and non-vegan vegetarians (*p* < 0.001).

Based on *K-means* cluster analysis, where all formed constructs (see Table 1) were taken into account, three consumer groups were formed. The three groups differed in their likelihood to consume foods of insect origin. “Likely consumers” exhibited the highest intention (Mean (M) 6.73) to consume insects in the future. Their attitude was the most positive (M 6.59), as well as their subjective norm (M 6.15). Further, they intended to consume foods of insect origin provided that they are nutritious (M 5.92), safe (M 6.33), affordable, and easy to prepare as food (M 6.32). “Potential consumers’” intention to use foods of insect origin was also high (M 5.50). Further, they were willing to eat insects if they are safe (M 4.7), convenient to use, and cheaper than meat (M 4.70). “Unlikely consumers” did not see themselves as insect food eaters in the future. Their intention to consume insects was low (M 1.65), even if insect foods were health-promoting and nutritious (M 1.42), safe (M 1.38), convenient to use, and cheaper than meat (M 1.38). Also, their perceived control over their eating was highest (M 6.02). According to the results of the Chi-Square Test (Table 6), most vegans (69.2%) were grouped into “unlikely consumers”, whereas 58.1% of non-vegan vegetarians, and 56.4% of omnivores, were grouped into “likely consumers.” Of non-vegan vegetarians, also 35.5% were classified into “potential consumers.” There were statistically significant differences in how the dietary groups were distributed into clusters of likely, potential, and unlikely consumers of insect foods (*p* < 0.001). 

## 4. Discussion

### 4.1. Attitudes and Planned Behavior toward Insect Consumption

Adherents of dietary groups (omnivores, non-vegan vegetarians, and vegans) differed significantly in their (A), (SN), and (PC). Consequently, also (I) to eat insects in the future differed significantly between the groups. Based on pairwise comparisons, vegans deviated significantly from both omnivores and non-vegan vegetarians in all studied constructs. By contrast, non-vegan vegetarians’ (I) to consume foods of insect origin did not deviate from that of omnivores, despite the fact that there was a significant difference in their attitude towards insect consumption (*p* < 0.05). This data, thus, partly confirms our hypothesis that vegans, non-vegan vegetarians, and omnivores differ from each other in their intention to eat foods of insect origin. Vegans’ weak (I) was expected, because they are known to deviate from non-vegan vegetarians in their food intake and food behavior (emotions, attitudes, and agency), as well as in their motives to adopt the diet, which all exert on their effects of food selection [11]. 

In particular, the conception of responsibility in food choice was an attitudinal aspect that made non-vegan vegetarians more similar to omnivores than to vegans. Vegans regarded themselves as responsible consumers while not eating foods of insect origin, which was the opposite of the other respondents (omnivores and non-vegan vegetarians), who regarded themselves as responsible consumers due to their eating insects because of their sustainability. 

Generally, vegans were significantly more determined than others in that they would not eat a food unless they knew the ingredients from which the food is made. They also proved to be significantly more selective when choosing their food. Vegans also felt more strictly that it is morally wrong to use insects in one’s own diet. This confirms our second hypothesis that vegans show the least willingness to consume foods of insect origin. It is very likely that vegans see fewer beneficial effects of insect consumption compared to the adherents of other dietary groups. Vegans are known to see greater moral problems with meat consumption, to be more disgusted by meat, and to have a more negative attitude toward meat-containing diets than do non-vegan vegetarians [7]. 

By possessing weak subjective norm (SN), i.e., less social pressure to eat insects, vegans appeared most independent in their food choices. However, they also highly appreciated the opinion of people close to them. This is consistent with earlier findings, showing vegans appreciating their peers’ opinions greatly [7,10,11]. 

Vegans also exhibited higher control over their food intake, which may be explained by food consumption strategies characteristic of vegans [10]. Vegans appeared significantly more neophobic than non-vegan vegetarians and omnivores, which may be partly explained by their food selection strategies and high perceived behavioral control over their food intake. However, it is also possible that vegans’ stricter moral attitude toward foods of animal origin lead them to eschew insects more strictly compared to non-vegan vegetarians. 

In the open responses, none of the vegans mentioned rejecting insect consumption due to disgust, despite the fact that in earlier studies vegans have been shown to have greater feelings of disgust toward meat. In vegans’ verbal justifications against insect consumption, moral and ethical reasoning seems to override potential feelings of disgust. Although ethics thus seems to make disgust irrelevant as a stated reason for rejecting insects as food, this does not necessarily mean that vegans are less disgusted by insects than others. 

### 4.2. Argumentation for Accepting or Rejecting Foods of Insect Origin

In addition to planned behavior and intention, we wanted to find out how adherents in different dietary groups argue for, or rationalize, their choices to either accept or reject foods of insect origin. When asking about reasons for consuming foods of insect origin, environmentalism, ecological reasons, and securing more sustainable futures were most often mentioned among omnivores and non-vegan vegetarians (data not shown). Characteristically, vegans cited being vegans, and omitting insects from their diet due to their ideology and moral reasons based on what they deemed best for the environment. Vegans did not consider foods of insect origin sustainable. For instance, it was noted that farming insects doesn’t help world hunger, and that cutting down food waste would be a more sustainable option for alleviating global nutritional problems.

Based on cluster analysis, most vegans (69%) were grouped into “unlikely consumers,” whereas 58% of non-vegan vegetarians and 56% of omnivores were “likely consumers.” Out of non-vegan vegetarians, 35% were classified into “potential consumers.” Unlikely consumers didn’t see themselves as insect food eaters in the future even if insect foods were health-promoting, nutritious, safe, convenient to use, and cheaper than meat. Modlinska and Pisula [28] have argued that persons who feel disgust against animal products are very likely to be the least willing to change their diet. We also know that vegans may deviate from each other in their compliance and agency towards veganism [14]. In our sample, every third vegan was clustered into either “likely” or “potential” consumers. Fischer [3] has argued that even strict vegans should eat insects provided that the insects are killed in a way that does not hurt them. However, our results show that so far, such an argument has not convinced vegans about the morality of insect consumption.

In conclusion, there were significant differences between the three dietary groups in all studied constructs (A, SN, PC, I, and FN). Non-vegan vegetarians were most positive towards insect consumption, while vegans were the least positive. Both omnivores and non-vegan vegetarians regarded insect consumption as wise and as a possibility in solving the world’s food problems. By contrast, vegans thought that insect consumption is irresponsible and morally wrong. 

Generalizing the results to the Finnish population entails a reservation due to convenience sampling, which has probably created a selection bias in terms of a more positive attitude toward insect consumption among the respondents compared to that of the population. Respondents were mostly women, highly educated, and city dwellers, a demographic profile known to impact food choice [33]. However, women are generally less eager compared to men to consume foods of insect origin [12,21,26]. In our population sample, the number of vegetarians (including both non-vegan vegetarians and vegans) proved to be relatively high (27%) compared to the estimates of the prevalence of vegetarianism in the Finnish population (4.1%) [34]. However, the benefit of this is that the number of non-vegan vegetarian and vegan respondents interested in insect consumption was large enough for us to detect some general characteristics of these groups. In the future, we need more research on dietarian identity profiles of consumer groups. In research on insect consumption, it would be valuable to study how people—both omnivores and different types of vegetarians—think, feel, and behave in their food choice, and how their dietary schemas [35] develop in time and during their dietarian “career”.

## Figures and Tables

**Table 1:** Constructs ^1^ M (SD) ^2^, Cronbach’s alphas, number of items, and examples of items.

Constructs	M (SD)	Alpha	Number of Items	Examples of Items
Intention	5.5 (2.0)	0.948	3	I intend to consume foods of insect origin when they are launched in Finnish markets.
				I am not going to consume foods of insect origin in any situation (R).
Attitude	5.7 (1.5)	0.970	11	The entering of foods of insect origin to the market would be a good trend.
				Using insects in food production is unnatural (R).
Subjective norm	5.4 (1.3)	0.898	8	People close to me probably find foods made from insects to be enjoyable.
				People important to me would worry if I ate foods of insect origin (R).
Perceived behavioral control	4.9 (1.3)	0.756	6	I watch what I eat carefully.
				I don’t know how to check whether my diet contains insects (R).
Healthiness	4.6 (1.9)	0.904	3	I intend to use foods of insect origin if they are nutritionally better than meat.
Safety	5.1 (1.9)	0.919	3	I intend to use foods of insect origin if they are found to be safe by health authorities.
Convenience/price	5.1 (1.9)	0.941	4	I intend to use foods of insect origin if they can be easily prepared as foods.
				I intend to use foods of insect origin if they are cheaper than meat.
Food neophobia	25 (11)	0.766	6	I constantly sample new and different foods (R).
				I don’t not trust new foods.
				If I don’t know what a food contains, I won’t try it.
				I try out new foods when I’m a food guest (R).
				I’m afraid to eat things I have never had before.
				I will eat almost anything (R).

R-reverse coded. ^1^ Likert scale (1–7) with response options 1 = totally disagree, 7 = totally agree. ^2^ Mean (Standard Deviation)

**Table 2:** Characteristics of the study participants (*n* = 567/%).

	Dietary Group			
	Omnivores (*n* = 417/73)	Non-Vegan Vegetarians ^1^ (*n* = 124/22)	Vegans (*n* = 26/5)	Significance ^2^
**Sex**				
Female (*n* = 379)	68	26	6	χ^2^ (2) = 14.753, *p* = 0.001
Male (*n* = 188)	84	14	2	
**Age**				
<25 (*n* = 73)	74	22	4	
25–29 (*n* = 119)	64	31	4	
30–39 (*n* = 143)	68	26	6	
40–49 (*n* = 112)	75	20	5	
>49 (*n* = 120)	88	8	4	χ^2^ (8) = 22.795, *p* = 0.004
**Education**				
Comprehensive school (*n* = 33)	73	15	12	
Senior high school/vocational school (*n* = 161)	80	16	5	
Academic degree (*n* = 334)	72	24	4	
Higher academic degree (*n* = 37)	59	38	3	χ^2^ (6) = 16.211, *p* = 0.002
**Place of residence**				
City area (*n* = 445)	70	25	5	
Rural area (*n* = 122)	86	12	2	χ^2^ (2) = 12.772, *p* = 0.002

^1^ The non-vegan vegetarian group includes semi-vegetarians (*n* = 97), lacto-vegetarians (*n* = 25), and lacto-ovo-vegetarians (*n* = 3). ^2^ Pearson Chi-Square test.

**Table 3:** One-Way ANOVA of the constructs of planned behavior ^1^ to eat foods of insect origin, and FN ^1^ in omnivores, non-vegan vegetarians and vegans (*n* = 567).

Construct	Omnivores (*n* = 417) M (SD)	Non-Vegan Vegetarians ^2^ (*n* = 124) M (SD)	Vegans (*n* = 26) M (SD)	Significance ^3^
Attitude (A)	5.69 (1.51)	6.11 (0.95)	3.71 (2.07)	F(2,564) = 29.941, ŋ^2^ = 0.096,
Subjective norm (SN)	5.42 (1.37)	5.70 (0.97)	4.20 (1.67)	F(2,564) = 14.049, ŋ^2^ = 0.047
Perceived behavioral control (PC)	4.80 (1.30)	4.81 (1.21)	5.93 (1.30)	F(2,564) = 9.655, ŋ^2^ = 0.033
Intention (I)	5.59 (1.95)	5.97 (1.54)	2.79 (2.55)	F(2,564) = 30.528, ŋ^2^ = 0.098
Food neophobia (FN)	26 (11)	27 (10)	31 (9)	F(2,707) = 5.131, ŋ^2^ = 0.018

^1^ Expressed as M (SD), 1–7 Likert scale with response options 1 = totally disagree, 7 = totally agree. ^2^ Non-vegan vegetarian group includes semi-vegetarians (*n* = 97), lacto-vegetarians (*n* = 25), and lacto-ovo-vegetarians (*n* = 3). ^3^ Mean values were significantly different between the dietary groups (One-Way ANOVA).

**Table 4:** One-Way ANOVA results of examples of significant differences toward insect consumption between dietary groups.

Variable *	Omnivores M (SD)	Non-Vegan Vegetarians M (SD)	Vegans M (SD)	Significance **
**Attitude**				
Insect consumption can be a solution to the world’s food problem.	5.57 (1.58)	5.93 (1.23)	3.96 (2.34)	F(2,564) = 16.913, ŋ^2^ = 0.056 a vs. c, *p* < 0.001 b vs. c, *p* < 0.001
Insects should not be used in food production.	1.86 (1.57)	1.45 (0.83)	4.19 (2.62)	F(2,564) = 35.655, ŋ^2^ = 0.112 a vs. c, *p* < 0.001 b vs. c, *p* < 0.001 a vs. b, *p* = 0.024
Using insects in food production is a good thing.	5.74 (1.66)	6.03 (1.31)	3.62 (2.42)	F(2,564) = 23.932, ŋ^2^ = 0.078 a vs. c, *p* < 0.001 b vs. c, *p* < 0.001
Foods made of insects are a bad thing.	1.98 (1.58)	1.63 (4.02)	4.15 (2.31)	F(2,564) = 29.614, ŋ^2^ = 0.033 a vs. c, *p* < 0.001 b vs. c, *p* < 0.001
The entering of foods of insect origin into the market would be a good trend.	5.54 (1.75)	6.11 (1.16)	3.42 (2.39)	F(2,564) = 28.073, ŋ^2^ = 0.090 a vs. c, *p* < 0.001 b vs. c, *p* < 0.001 a vs. b, *p* = 0.003
The use of insects should be promoted in food production.	5.62 (1.74)	6.06 (1.14)	3.35 (2.62)	F(2,564) = 28.120, ŋ^2^ = 0.091 a vs. c, *p* < 0.001 b vs. c, *p* < 0.001 a vs. b, *p* = 0.034
The practice of eating of insects is wise.	5.34 (1.64)	5.73 (1.40)	3.31 (2.36)	F(2,564) = 23.840, ŋ^2^ = 0.078 a vs. c, *p* < 0.001 b vs. c, *p* < 0.001
The use of insects as human food should definitely be approved in Finland.	5.85 (1.67)	6.32 (1.18)	4.15 (2.48)	F(2,564) = 19.358, ŋ^2^ = 0.064 a vs. c, *p*< 0.001 b vs. c, *p* < 0.001 a vs. b, *p* = 0.014
The use of insects in food production is morally wrong.	1.75 (1.34)	1.84 (1.33)	5.27 (2.07)	F(2,564) = 80.637, ŋ^2^ = 0.222 a vs. c, *p* < 0.001 b vs. c, *p* < 0.001
I want to be an ethically responsible consumer and I don’t want to utilize insects in my own diet.	2.27 (1.83)	2.32 (1.81)	5.42 (2.40)	F(2,564) = 35.709, ŋ^2^ = 0.112 a vs. c, *p* < 0.001 b vs. c, *p* < 0.001
I want to be a responsible consumer and eat insects since I know that eating them is sustainable.	5.22 (1.92)	5.44 (1.62)	2.69 (2.22)	F(2,564) = 24.009, ŋ^2^ = 0.078 *p* < 0.001
**Subjective norm**				
It is important to me to fulfill other people’s expectations.	2.25 (1.44)	2.68 (1.61)	1.65 (1.06)	F(2,564) = 6.813, ŋ^2^ = 0.023 a vs. b, *p* = 0.015 b vs. c, *p* = 0.004
People I respect could eat foods of insect origin.	5.68 (1.58)	6.22 (1.10)	5.04 (2.04)	F(2,564) = 9.157, ŋ^2^ = 0.031 a vs. b, *p* = 0.002 b vs. c, *p* = 0.001
I could eat foods of insect origin since people close to me think that I follow novel trends in my food selection.	3.18 (1.83)	3.63 (1.79)	2.15 (2.09)	F(2,564) = 7.588, ŋ^2^ = 0.026 a vs. c, *p* = 0.017 b vs. c, *p* = 0.001
I could eat foods of insect origin if my friends recommended eating them.	5.25 (1.95)	5.52 (1.66)	2.50 (2.30)	F(2,564) = 27.894, ŋ^2^ = 0.090 a vs. c, *p* < 0.001 b vs. c, *p* < 0.001
People that are important to me would respect me less if I ate foods made of insects.	1.78 (1.38)	1.56 (1.11)	3.3 (2.24)	F(2,564) = 17.597, ŋ^2^ = 0.059 a vs. c, *p* < 0.001 b vs. c, *p* < 0.001
**Perceived behavioral control**				
I can easily control that my diet doesn’t contain insects.	4.50 (2.10)	4.23 (2.14)	5.73 (1.80)	F(2,564) = 5.491, ŋ^2^ = 0.019 a vs. c, *p* < 0.001 b vs. c, *p* < 0.001
I always watch what I eat carefully.	4.63 (1.74)	4.64 (1.55)	6.19 (1.68)	F(2,564) = 10.733, ŋ^2^ = 0.036 a vs. c, *p* < 0.001 b vs. c, *p* < 0.001
It is totally up to me whether I buy foods made of insects.	6.02 (1.46)	5.66 (1.55)	6.65 (1.06)	F(2,564) = 5.806, ŋ^2^ = 0.020 b vs. c, *p* = 0.005
**Food neophobia**				
If I don’t know what the food contains, I won’t try it.	3.9 (2.07)	4.0 (1.98)	6.04 (1.69)	F(2,564) = 13.404, ŋ^2^ = 0.045 a vs. c, *p* < 0.001 b vs. c, *p* < 0.001
I am very selective in what I eat.	3.11 (1.83)	3.67 (1.82)	4.31 (2.04)	F(2,564) = 8.607, ŋ^2^ = 0.036 a vs. c, *p* = 0.004 a vs. b, *p* = 0.009
I will eat almost anything.	5.03 (1.89)	3.92 (1.47)	3.0 (1.92)	F(2,564) = 26.995, ŋ^2^ = 0.087 a vs. b, *p* < 0.001 a vs. c, *p* < 0.001

* 1–7, Likert scale, response options (1 = totally disagree, 7 = totally agree). ** a = omnivores, b = non-vegan vegetarians, c = vegans.

**Table 5:** One-Way ANOVA of the Perceived behavioral condition ^1^ to consume foods of insect origin among omnivores, non-vegan vegetarians, and vegans (*n* = 567).

Construct	Omnivores (*n* = 417) M (SD)	Non-Vegan Vegetarians ^2^ (*n* = 124) M (SD)	Vegans (*n* = 26) M (SD)	Significance ^3^
Healthiness	4.69 (1.88)	4.92 (1.53)	2.40 (2.00)	F(2,564) = 21.476, ŋ^2^ = 0.071
Safety	5.04 (1.91)	5.58 (1.38)	2.60 (2.32)	F(2,564) = 28.351, ŋ^2^ = 0.091
Convenience and price	5.13 (1.90)	5.33 (1.40)	2.38 (2.04)	F(2,564) = 29.913, ŋ^2^ = 0.096

^1^ Expressed as M (SD), 1–7 Likert scale with response options 1 = totally disagree, 7 = totally agree. ^2^ The non-vegan vegetarian group includes semi-vegetarians (*n* = 97), lacto-vegetarians (*n* = 25), and lacto-ovo-vegetarians (*n* = 3). ^3^ Mean values were significantly different between dietary groups (One-Way ANOVA); *p* < 0.001.

**Table 6:** Cross-tabulation and Chi Square test of Division of dietary groups into likely, potential, and unlikely consumers based on their planned behavior and perceived behavioral conditions (*n* = 567) ^1^.

	Likely Consumers (*n* = 311, 55%)	Potential Consumers (*n* = 163, 29%)	Unlikely Consumers (*n* = 93, 16%)	Total	Significance ^2^
Dietary group (*n*, %)					
Omnivores (*n* = 417, 73)	56.4	27.6	16.1	100.0	χ^2^ (4) = 62.315 0.234 (Cramer’s V)
Non-Vegan Vegetarians (*n* = 124, 22)	58.1	35.5	6.5	100.0	
Vegans (*n* = 26, 5)	15.4	15.4	69.1	100.0	

^1^ Scale means (range 1–7) based on 1–7 Likert scale with response options 1 = totally disagree, 7 = totally agree. ^2^ Pearson Chi-Square test, *p* < 0.001.
